# Supplementary material for: HIVprotI: an integrated web based platform for prediction and design of HIV proteins inhibitors
Source: J Cheminform. 2018 Mar 9;10:12. doi: 10.1186/s13321-018-0266-y (PMC5845081; doi:10.1186/s13321-018-0266-y)
Supplement: Supplementary file 2 — Additional file 2. Source code of HIVProtI web server. [file 13321_2018_266_MOESM2_ESM.zip › HIVprotI_Source-code/hivprotI/weka-3-6-6/doc/weka/classifiers/trees/package-summary.html]

weka.classifiers.trees


---


|  |  |  |
| --- | --- | --- |
| **Overview** \| **Package** \| Class \| **Tree** \| **Deprecated** \| **Index** \| **Help** \| **Weka's home** | |  |
| **PREV PACKAGE**   **NEXT PACKAGE** | **FRAMES**    **NO FRAMES**     **All Classes** |  |


---

## Package weka.classifiers.trees

| **Class Summary** | |
| --- | --- |
| **ADTree** | Class for generating an alternating decision tree. |
| **BFTree** | Class for building a best-first decision tree classifier. |
| **DecisionStump** | Class for building and using a decision stump. |
| **FT** | Classifier for building 'Functional trees', which are classification trees that could have logistic regression functions at the inner nodes and/or leaves. |
| **Id3** | Class for constructing an unpruned decision tree based on the ID3 algorithm. |
| **J48** | Class for generating a pruned or unpruned C4.5 decision tree. |
| **J48graft** | Class for generating a grafted (pruned or unpruned) C4.5 decision tree. |
| **LADTree** | Class for generating a multi-class alternating decision tree using the LogitBoost strategy. |
| **LMT** | Classifier for building 'logistic model trees', which are classification trees with logistic regression functions at the leaves. |
| **M5P** | M5Base. |
| **NBTree** | Class for generating a decision tree with naive Bayes classifiers at the leaves.    For more information, see    Ron Kohavi: Scaling Up the Accuracy of Naive-Bayes Classifiers: A Decision-Tree Hybrid. |
| **RandomForest** | Class for constructing a forest of random trees.    For more information see:     Leo Breiman (2001). |
| **RandomTree** | Class for constructing a tree that considers K randomly chosen attributes at each node. |
| **REPTree** | Fast decision tree learner. |
| **SimpleCart** | Class implementing minimal cost-complexity pruning.  Note when dealing with missing values, use "fractional instances" method instead of surrogate split method.    For more information, see:    Leo Breiman, Jerome H. |
| **UserClassifier** | Interactively classify through visual means. |

---


|  |  |  |
| --- | --- | --- |
| **Overview** \| **Package** \| Class \| **Tree** \| **Deprecated** \| **Index** \| **Help** \| **Weka's home** | |  |
| **PREV PACKAGE**   **NEXT PACKAGE** | **FRAMES**    **NO FRAMES**     **All Classes** |  |


---
